# Metal Profiles in Autism Spectrum Disorders: A Crosstalk between Toxic and Essential Metals

**DOI:** 10.3390/ijms24010308

**Published:** 2022-12-24

**Authors:** Anna Błażewicz, Andreas M. Grabrucker

**Affiliations:** 1 Department of Pathobiochemistry and Interdisciplinary Applications of Ion Chromatography, Medical University of Lublin, 20-093 Lublin, Poland; 2 Department of Biological Sciences, University of Limerick, V94 T9PX Limerick, Ireland; 3 Bernal Institute, University of Limerick, V94 T9PX Limerick, Ireland; 4 Health Research Institute (HRI), University of Limerick, V94 T9PX Limerick, Ireland

**Keywords:** cadmium, lead, mercury, oxidative stress, lipid peroxidation, ASD, mitochondria, inflammation, zinc, copper

## Abstract

Since hundreds of years ago, metals have been recognized as impacting our body’s physiology. As a result, they have been studied as a potential cure for many ailments as well as a cause of acute or chronic poisoning. However, the link between aberrant metal levels and neuropsychiatric illnesses such as schizophrenia and neurodevelopmental disorders, such as autism spectrum disorders (ASDs), is a relatively new finding, despite some evident ASD-related consequences of shortage or excess of specific metals. In this review, we will summarize past and current results explaining the pathomechanisms of toxic metals at the cellular and molecular levels that are still not fully understood. While toxic metals may interfere with dozens of physiological processes concurrently, we will focus on ASD-relevant activity such as inflammation/immune activation, mitochondrial malfunction, increased oxidative stress, impairment of axonal myelination, and synapse formation and function. In particular, we will highlight the competition with essential metals that may explain why both the presence of certain toxic metals and the absence of certain essential metals have emerged as risk factors for ASD. Although often investigated separately, through the agonistic and antagonistic effects of metals, a common metal imbalance may result in relation to ASD.

## 1. Introduction

Difficulties in social communication, constrained and focused interests, speech deficits, and language delays are the three main characteristics of ASD. The Diagnostic and Statistical Manual of Mental Disorders, Fifth Edition, states that the first two characteristics are utilized to identify ASD. Further signs and conditions in people with ASD include intellectual disability, anxiety, depression, attention deficit disorder, hyperactivity, impulsivity, seizures, gastrointestinal issues, sensory dysfunction, aggression, metabolic disorders, sleep disorders, motor dysfunction, and altered immune responses [1]. The intensity of the symptoms and comorbidities varies, leading to significant variability in the clinical characteristics of people with ASD.

ASD is officially recognized in 1 in 54 children in the United States, according to data from the Centers for Disease Control and Prevention Agency. Due to changes in diagnostic criteria, outcomes of European research have varied over time, and numbers vary from 1 in 210 to 1 in 32. In Asia, rates between 1 in 322 and 1 in 20 have been reported [1].

The history of ASD and the quest for potential explanations are closely related to the history of metals as a risk factor for ASD. Since Leo Kanner and Hans Asperger, it has been debated how much genetic and environmental variables contribute to ASD. Recent developments in genetics, particularly in DNA sequencing technology, have brought more attention to genetic aspects. The Simons Foundation Autism Research Initiative (SFARI) gene database lists 1095 identified ASD-related genes in several categories, reflecting the strength of the evidence linking the gene to ASD as of October 2022. Notably, the encoded proteins of some ASD candidate genes, such as Copper Metabolism Domain Containing 1 (*COMMD1*), Metal Regulatory Transcription Factor 1 (*MTF1*), and Zinc Transporter 5 (*SLC30A5*), play a role in metal transport [2].

Contrarily, throughout decades, only a small number of conclusive environmental factors of ASD have been found [2]. Thus, these factors either account for the majority of ASD cases without a known genetic cause or the identification of environmental factors is slower because research tools and techniques are lacking. As a result, there is still conflicting information regarding the role that hereditary and environmental variables play in ASD. However, regardless of the underlying causes, people with ASD consistently exhibit the three main symptoms. Therefore, it is likely that hereditary and environmental factors contribute to ASD by impacting a single putative brain mechanism. According to the available data, this neurological pathomechanism in ASD is linked to abnormal synaptic processes [2]. 

Over the past ten years, many studies have reported increased ASD diagnosis. For instance, research on autism conducted in the 1960s in Europe and the US suggested that ASD was an uncommon condition with an incidence of 2 to 4 children per 10,000 [1]. Albeit there may be a correlation between the increase in diagnoses and more awareness and a shift in diagnostic standards, environmental factors, while poorly understood, may also have a role.

In light of this, the link between ASD and heavy metal pollution was already discussed in the late 1970s, with studies reporting elevated lead (Pb) levels in autistic children [3]. Heavy metal overload was among the first non-genetic factors investigated in ASD research. However, few early studies considered the developmental time window of exposure that appears crucial for an environmental factor to function as a risk factor for ASD. Many non-genetic variables, if not all, act during in-utero exposure [4]. Epidemiological studies on metals are affected by the difficulty of measuring lack or exposure to certain metals retrospectively during pregnancy. This may also explain the mixed results of studies measuring older individuals with ASD since a brief exposure or deficiency during pregnancy may no longer be detected, and no biosamples (e.g., blood, urine) from the mother during pregnancy are available.

However, early epidemiological data that suggested the involvement of toxic heavy metals in the etiology of ASD provided the first supporting evidence for “heavy metal pollution” as a contributing factor. While the definition of a “heavy metal” is not always clear, concerning ASD and human health, “heavy metal” generally refers to the presence of abnormally high concentrations of the toxic metals lead (Pb), nickel (Ni), cadmium (Cd), and mercury (Hg). These studies formed the basis of the hypothesis that ASD may develop due to prenatal exposure to heavy metals [5]. More recently, besides correlations between elevated levels of copper, lead, mercury, and cadmium [6], circumstances associated with increased exposure to those metals have been linked to ASD, such as living close to gas stations or industrial facilities [7,8]. Thus, research data on metals in disease etiology fit into the concept of an exposome, proposed by Wild, defining a broad approach to the environmental component to improve and understand predictors and risk factors for multifactorial pathologies such as ASD [9]. According to the literature, “the exposome represents the totality of exposures from conception onwards, simultaneously identifying, characterizing, and quantifying the exogenous and endogenous exposures and modifiable risk factors that predispose to and predict diseases throughout a person’s life span” [10]. The combination of the biological effects of metals in tissues and biofluids with data from multiple exposures is a complex task. However, it complements our genome, defining our innate predisposition to certain diseases. Literature highlights that exposome studies may create opportunities to prioritize the more relevant chemicals for risk assessment [11]. 

The increasing number of studies on ASD and metals uses classical exposure science linking precise measurement of single or multiple exposures related to outcomes of disorders. However, more and more of them emphasize the importance of combining exposure data with lifestyle and social factors [12]. Hair, nails, and tooth samples offer historical data on metal exposure, whereas blood and urine samples measure acute exposure. Particularly the enamel of baby teeth can provide evidence of prenatal and early postnatal exposure as the formation of primary teeth begins during pregnancy, and they fully grow between three months and a year after delivery [13]. Numerous investigations using different types of samples have revealed that children with ASD have greater levels of toxic metals in their bodies than neurotypical controls [14,15,16,17,18,19,20,21,22,23,24,25,26,27,28,29,30,31,32,33,34,35,36,37,38,39] (Table 1). In some studies, the degree of ASD severity correlates with a heavy metal load. Table 1 provides examples of epidemiological studies investigating the association of toxic metals with ASD. The studies listed utilize different designs, methods of case determination, biomarkers, statistical analysis, and biosamples and vary in participant numbers, resulting in variable evidence strength. For a systematic review and meta-analysis with defined inclusion criteria, see [38,39].

## 2. Heavy Metal Overload and ASD

### 2.1. Lead

Prenatal lead exposure can result from lead that has built up in the mother’s bones from previous exposures as well as acute maternal exposure. The main causes of lead exposure in modern times include lead dust from deteriorated lead-based paint, lead in drinking water due to leaching from lead-containing pipes in older buildings’ plumbing, polluted soil near businesses where lead is or was utilized, and lead from exposure to cigarette smoke. However, some commercial goods, such as jewelry, kid’s toys, and cosmetics, may also contain lead [40]. Lead affects the central nervous system (CNS), even in trace amounts. Its presence causes many clinical symptoms, including problems in cognition, attention, memory, and behavior [41,42]. While the absolute levels of lead found in biosamples of children have fallen over the recent years due to the ban on leaded fuel, children with ASD are reported consistently to show higher blood levels of lead [15,16,21,22,38,43,44,45]. Given that higher levels were found in the nail and hair but also in blood samples, the findings imply that children with ASD had greater levels of both recent and long-term exposures. However, a comprehensive metallomics investigation utilizing hair samples from children with ASD indicated that the youngest group (infants aged 0–3 years) had the highest lead burden, suggesting a link between early perinatal exposure and ASD [23]. However, some studies failed to identify a connection between lead levels and the prevalence of ASD [25,26]. A recent meta-analysis that included the findings of 48 independent research studies discovered substantially higher lead hair concentrations in ASD patients compared to controls [38].

### 2.2. Mercury

Even though many nations have rules and procedures in place to reduce exposure to it, mercury—which may exist as both inorganic (elemental) mercury and organic mercury—is a metal that is prevalent in our environment. Mainly organic mercury, such as methylmercury (MeHg), which humans often absorb through food, such as freshwater and marine fish, is to blame for human exposure to mercury [46,47]. ASD has been linked to higher mercury levels, according to many studies [16,19,22,27], with an emphasis again on the pre-and early postnatal developmental period. For example, the primary teeth of children with ASD show considerably greater mercury amounts [26]. However, no significant differences were discovered when measuring enamel that is created later in life [48]. Additional reports of elevated mercury levels and increased urine porphyrin excretion, a biomarker of mercury poisoning, were published using blood samples from children with ASD [17,18,28,29,49,50,51].

### 2.3. Cadmium

Several human activities, including the burning of garbage and the usage of phosphate fertilizers, release cadmium into the environment [52], which may get enriched in meat, fish, vegetables, and fruits as a result of high amounts of the metal in the soil and water supplies [53]. Additionally, exposure to cigarette smoke may allow cadmium to enter the body [54]. Several studies indicate a link between high cadmium levels and ASD [27,30,31]. For example, according to a metallomics investigation, 8.5% of ASD children aged 0 to 15 exhibited elevated cadmium loads in hair samples [24]. The largest body load was again seen in babies aged 0–3 years [23,24].

It is important to remember that some toxic metals reach the environment through similar processes, such as trash incineration. Thus, excessive mercury exposure, lead, and cadmium are frequently co-occurring. Studies that measure multiple metals in parallel confirm that, for instance, those with high levels of lead also have high amounts of cadmium. Therefore, rather than each harmful metal by itself, the mixture of these metals could increase the risk of ASD. The impacts of these metals may work cumulatively since co-exposure may influence neurodevelopment through similar pathways.

## 3. Pathomechanisms of Heavy Metal Overload in ASD

Toxic metals may impact brain development directly, as they are able to reach the brain tissue. For example, MeHg can readily pass through the placenta and the blood-brain barrier (BBB). Therefore, it is more neurotoxic than inorganic mercury, which moves through membranes more slowly [55]. Besides, inorganic mercury, such as lead, can damage the BBB and thus allow entry into the brain parenchyma, for example, through the dysregulation of astrocytes that are part of the BBB structure. Cadmium may circumvent the BBB and enter the brain via the olfactory route, notably in cigarette smokers. However, cadmium may also affect BBB integrity. In addition, transport proteins that regulate the homeostasis of essential trace metals may be used by toxic metals. For instance, although the divalent metal transporter (DMT)1 prefers iron as a substrate under physiological conditions, it can bind and transport a variety of metals, including lead and cadmium, across membranes [56].

It should be noted that during early brain development, the BBB is not fully functional yet. Thus, a developing brain may be more susceptible to the crossing of toxic metals into brain tissue. Besides, toxic metals such as lead may directly impede BBB formation [57]. Furthermore, toxic metals may not even have to reach the brain, as they might affect brain development indirectly by causing an extracerebral pathology that affects the brain, i.e., through gut-brain signaling, etc. 

The consequences of the interferences of toxic metals for brain development, whether as an effect of the metal’s acute presence in the brain or as a secondary effect of peripheral pathologies affecting the brain, are difficult to model and investigate. Toxic metals may interfere with dozens of physiological processes. Nevertheless, the pathomechanisms of toxic metals have been somewhat elucidated; those pertinent to ASD will be addressed in this review.

**Figure 1:**
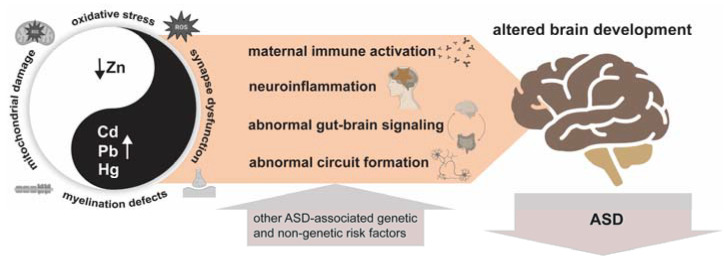
Effects of toxic trace metals: One characteristic of high levels of mercury, lead, and cadmium is that they compete with essential trace metals such as zinc, thereby affecting neurogenesis and neural differentiation, synapses, myelination, and inflammatory processes. Additionally, toxic metals worsen mitochondrial dysfunction and raise oxidative stress, resulting in lipid peroxidation, poor energy, and cell death. By harming the blood-brain barrier or activating astrocytes and microglia, toxic metals also have direct proinflammatory effects that may cause gliosis. Besides, an abnormal metal profile will affect the gastrointestinal system and its microbiota, leading to abnormal gut-brain signaling. Ultimately, the impacted processes will lead to an altered brain development through similar mechanisms proposed for other genetic and non-genetic risk factors for ASD.

There are several similarities between the cellular pathologies associated with ASD and those caused by mercury, lead, and cadmium overdose: The competition with essential metals, especially zinc, oxidative stress, lipid peroxidation, mitochondrial dysfunction, neuroinflammation and gliosis, and axonal demyelination are generally the main pathologies seen after exposure to high levels of toxic metals. The affected processes are highly active in the developing brain (Figure 1). Toxic metals may interfere with normal brain development through the abovementioned processes by various mechanisms, such as DNA methylation, histone modifications, microRNA expression, changing protein properties, or affecting gut-brain signaling by altering the microbiota composition [58]. The ASD-related effects may only occur during a person’s prenatal period. However, certain consequences, such as a gene or protein expression change, might not manifest until later in life [59]. Especially early development is a critical window of susceptibility since the placenta is ineffective at preventing the passage of several toxic metals to the fetus [57], and the embryo’s blood-brain barrier (BBB) is still developing [60].

### 3.1. Oxidative Stress and Mitochondrial Dysfunction

Elevated levels of oxidative stress are a well-described feature of ASD [61,62,63,64,65]. The actions in mitochondria that result in the synthesis of adenosine triphosphate (ATP) naturally produce free radicals, also known as reactive oxygen species (ROS). Reactive nitrogen species (RNS) and radicals with carbon and sulfur centers are also produced [66]. As a result, small amounts of ROS in brain tissue should be regarded as normal, but large amounts that cause oxidative stress are harmful. Excessive free radical generation or insufficient cellular mechanisms to alleviate oxidative stress can contribute to elevated oxidative stress levels. The brain is particularly vulnerable to oxidative stress because of its high energy (ATP) requirements.

The brain is a lipid-rich tissue. Lipid peroxidation thus poses a particular threat to brain cells [66]. However, excess ROS also causes protein oxidation in addition to lipid peroxidation. Together, the two processes might be responsible for aberrant neurogenesis, synaptic signaling, plasticity, brain cell death, and functional deficits [67,68,69,70]. For example, oxidative stress may change brain-derived neurotrophic factor levels (BDNF) [71]. BDNF is a growth factor linked to the pathogenesis of ASD as a regulator of neurogenesis, neuron survival, and synaptic plasticity [72]. 

As underlying reasons for increased ROS in ASD, numerous studies implicate an increase in BBB permeability that results in neuroinflammation [73,74]. For example, it has been noted that exposure to mercury damages the BBB, exposing the CNS to undesired substances that might cause processes such as neuroinflammation [75]. Additionally, it has been suggested that excitotoxicity caused by aberrant glucocorticoid receptor signaling, and altered N-methyl-d-aspartate (NMDA) receptor signaling contribute to ROS [76,77,78].

One defense mechanism that can lower ROS levels is the antioxidant response system. It works by activating antioxidant enzymes such as catalase (CAT), glutathione peroxidase, glutathione reductase, glyoxalase, and superoxide dismutase (SOD) [79]. Interestingly, SOD enzymes require essential trace metals, for example, copper and zinc (Cu-Zn SOD). Low-molecular-weight antioxidants such as glutathione, uric acid, ascorbic acid, and melatonin are used in the second stage of the antioxidant response system. These primarily act through scavenging metals [80].

Elevated levels of oxidative stress are also a well-described feature of heavy metal exposure. Mercury is one of the toxic metals that raises oxidative stress levels and harms mitochondria [81,82,83]. Mercury can inhibit sulfhydryl-containing enzymes by forming a covalent bond with sulfhydryl (thiol) groups [84]. Similarly, other proteins and non-protein molecules, such as glutathione (GSH), may be impacted by the direct chemical interaction between MeHg and thiol [84]. Due to GSH’s function as an antioxidant, if GSH levels fall, oxidative stress rises [85]. However, it has also been demonstrated that MeHg directly interacts with nucleophilic protein groups to cause oxidative stress [79]. In addition, mercury exposure also results in glutamate excitotoxicity [86]. MeHg increases glutamate levels in the synaptic cleft by inhibiting glutamate re-uptake into synaptic vesicles and promoting spontaneous release, according to in vitro and animal studies [86,87,88,89]. 

Abnormally high glutamate concentrations in the synaptic cleft can cause NMDA-type glutamate receptor (R) overactivation. Apart from direct consequences on synapse formation, stability, and function, this raises the amount of sodium and calcium that enters neurons through NMDARs. In response, excessive intracellular calcium will again boost oxidative stress (for example, by translocation into mitochondria) [90]. Because astrocytes help remove the glutamate from the synaptic cleft, mercury injury to astrocytes exacerbates excitotoxicity [91].

Excessive cadmium concentrations also result in oxidative stress. Due to its affinity for thiol groups, cadmium may accumulate inside cells as cadmium-thiol complexes [92]. As a result, GSH levels fall, impacting cellular defenses against ROS [93]. Additionally, cadmium interferes with cellular protein breakdown, which is demonstrated by an increase in the number of ubiquitinated proteins. As a result, cadmium disrupts many cellular functions, including the antioxidant response [94,95]. Besides, ROS production by lead has been identified as a key mechanism underpinning lead poisoning. For example, the δ-aminolevulinic acid (ALA) dehydrase (ALAD) is affected by the presence of lead, leading to elevated ALA levels, which induces the production of ROS. The oxidation product of ALA may, in turn, cause DNA damage [96]. In addition, lead impacts the antioxidant defense mechanisms [97]. By blocking functional sulfhydryl groups in various enzymes, including SOD, CAT, glutathione peroxidase, and glucose-6-phosphate dehydrogenase, lead has been demonstrated to change antioxidant activity [97].

Oxidative stress and mitochondrial damage are intimately related. Thus, mitochondrial dysfunction may be linked to ASD [98,99,100,101,102,103]. Mitochondria oxidize glucose and fatty acids to produce ATP. In addition, the mitochondria play critical roles in calcium homeostasis and programmed cell death (apoptosis). They impact CNS neuronal functions such as synaptic plasticity and neurotransmitter release [104]. Brain cells are cells with a high energy requirement. Therefore, they have more mitochondria and are particularly vulnerable. 

Children with ASD may have mitochondrial (mt) damage, which manifests as mtDNA over-replication, mtDNA deletions, and mitochondrial malfunction (lower mitochondrial-dependent oxygen consumption and increased hydrogen peroxide (H_2_O_2_) generation) [98,105]. It was reported that symptoms discovered as a result of mitochondrial malfunction coincide with those of ASD, suggesting that mitochondrial damage may contribute to the phenotype of ASD [106]. A reduced synaptic neurotransmitter release and improper calcium signaling due to mitochondrial malfunction have been proposed as potential pathomechanisms. Notably, plasma membrane calcium channels are impacted by mitochondria, which serve as calcium reserves [107].

### 3.2. Neuroinflammation

Numerous immunological disorders can arise from toxic metal exposure. In epidemiological research and mechanistic studies utilizing animal models, disorders of the immune system and inflammation have been linked to ASD. It has been demonstrated that lead, cadmium, and mercury directly influence gene expression. The altered genes included those that encode proteins key to regulating oxidative stress and inflammation, among others [108,109,110]. Furthermore, it was shown that lead and methylmercury could directly trigger glial reactivity [111], and a rise in ROS and BBB permeability may be a component of this. For example, mercury is concentrated in astrocytes and, to a lesser extent, in microglial cells inside the CNS [112,113], which causes these cells to become dysfunctional [114]. Notably, activated glial cells such as microglia and astrocytes release the proinflammatory cytokines Tumor Necrosis Factor (TNF), Interleukin (IL)-1, and IL-6 [115]. TNF, IL-1, and IL-6 are elevated in biosamples of individuals with ASD, and their levels positively correlate with ASD severity [116,117,118]. Animal studies have demonstrated that IL-6, in particular, may change synapse formation and plasticity [119,120]. 

In susceptible people, elevated mercury and lead levels result in increased production of proinflammatory cytokines and the development of autoantibodies even at low levels of chronic exposure [121,122,123]. For instance, one study discovered a correlation between increased blood levels of autoantibodies and increased mercury levels [124]. Neuronal cytoskeletal proteins, neurofilaments, and myelin basic protein (MBP) are among the proteins that lead and mercury-induced autoantibodies target [125].

### 3.3. Axonal Demyelination

Numerous studies have linked ASD to white matter abnormalities, including hypomyelination [126,127,128]. Neuronal axons in the CNS are myelinated when oligodendrocytes create myelin, a multi-lamellar membrane rich in lipids. Myelination is important in establishing connectivity in the developing brain. Toxic trace metals affect myelination. For example, lead exposure has been connected to disrupted connectivity [129,130]. Glial cells, including oligodendrocytes, take up lead [131,132]. A reduction in the activity of 2′,3′-Cyclic-nucleotide 3’-phosphodiesterase (CNPase) by lead, an enzyme that has been demonstrated to be essential for myelin formation, might be a factor explaining reduced myelinization [133]. The CNS white matter is also damaged by cadmium. It was shown that oligodendrocytes, and more so oligodendrocyte progenitors, are susceptible to cadmium. Cadmium toxicity was tied to excessive production of ROS, leading to oligodendrocyte death or dysfunction. In addition, MeHg toxicity is linked to the downregulation of MBP in pregnant and lactating rats [134]. MBP is essential for the construction and function of the myelin sheaths by oligodendrocytes and Schwann cells.

### 3.4. Competition with Essential Metals, Especially Zinc

By competing with essential metals such as calcium and zinc for protein binding, toxic metals can affect activities mediated by these metals. In particular, calcium and zinc are critical intracellular signaling ions bound by many proteins. For example, around 10% of the human genome encodes for zinc-binding proteins [135]. Zinc and lead may compete for the same protein binding sites [136,137]. Zinc and mercury were also discovered to interact, and cadmium can inhibit the transport of zinc and calcium, including the transfer of zinc to the fetus [138]. In turn, this will influence the activity of enzymes and second messengers and interfere with cellular signaling, transport, and metabolism. Additionally, cadmium should compete somewhat with magnesium, copper, and iron [139]. The disturbance of physiological processes mediated by metals, such as calcium and zinc, may be a major pathomechanism of toxic metals [140] and contribute to the abovementioned processes.

For example, myelination is a zinc-dependent process. A study on rhesus monkeys showed that maternal zinc deficiency causes the offspring’s myelin protein profiles to change [141]. Additionally, it was discovered that MBP, a target of autoantibodies generated by lead and mercury, is a zinc-binding protein, indicating a function for zinc in myelin compaction [142,143]. Further, in addition to promoting the production of free radicals, toxic metals, such as cadmium, lead, and mercury, may also compromise the antioxidant system by displacing essential metals from antioxidant enzymes such as Cu-Zn SOD. Lead also disrupts mitochondrial calcium and substitutes calcium in calcium signaling systems, altering Calmodulin and numerous downstream protein kinases [144]. Thereby, lead impedes synaptic signaling and vital cellular processes, such as proliferation and differentiation, for example, by changing the activity of the protein kinase C (PKC) enzyme. Lead also reduces the calcium-dependent release of neurotransmitter vesicles [145]. This can be a way that lead affects glutamatergic neurotransmission [146], which is also dependent on critical zinc-regulated synaptic proteins [147]. 

Given that zinc can be replaced or the levels of zinc lowered by toxic metals, some of the consequences of zinc deficiency may be imitated by toxic metal overload. Thus, prenatal zinc deficiency and heavy metal overload may be two sides of the same coin. 

## 4. Toxic Metals Causing a Zinc Deficiency—A Model for the Convergence of Abnormal Trace Metal Levels in ASD

The essentiality of zinc—the second-most common trace metal present in our body—was first established in 1963. Before zinc, all other and even less common essential trace metals (i.e., iron in 1932, copper in 1928, manganese in the 1950s, and cobalt in 1948 when it was discovered that it is a component of vitamin B-12) were identified. However, zinc is now the main topic of discussion concerning ASD, as zinc insufficiency is a repeating theme established in several meta-analyses [148,149,150]. Most research evaluating zinc in people with ASD utilizes hair zinc levels; hair is the more dependable biosample for zinc than blood or urine, given that zinc levels in plasma may fluctuate by as much as 20% over one day, creating a challenge to detect mild zinc deficiencies in particular [151]. While few studies find no changes between the zinc levels of the ASD group and controls, the majority report noticeably lower zinc levels. Age and zinc levels were correlated, with the youngest participants with ASD having the greatest incidence rate of zinc insufficiency (43.5% in males and 52.5% in females), according to the perhaps most comprehensive study to date that examined 1,967 hair samples of children with ASD [152].

Zinc is essential for neurodevelopment, and altered zinc status is implicated in various ASD animal models based on the manipulation of hereditary and non-genetic variables. For example, maternal immune activation (MIA) in rats during pregnancy results in offspring with ASD-like behavioral abnormalities and low zinc levels [153]. Therefore, MIA mice and rats may behave similarly to prenatally zinc-deficient animals. Prenatal zinc therapy averted these behavioral issues linked to ASD [154,155]. On the cellular level, the mammalian target of rapamycin (mTOR) levels were abnormal in the striatum in response to MIA, and elevated levels of BDNF were found. Zinc supplementation throughout pregnancy also normalized mTOR and BDNF levels [154,155]. Further, rodents exposed to valproic acid (VPA) during pregnancy have ASD-like characteristics [156]. Exposure to VPA may change how zinc is metabolized, causing transient zinc deficiency [157]. Therefore, as was the case with MIA mice, the changed zinc status of the pregnant mice may be what is causing the ASD-like behavior. Indeed, prenatal zinc therapy decreased VPA-induced ASD-like behaviors [157].

Zinc deficiency alone during pregnancy causes ASD-like behavior in mice [158]. This is an important finding as it supports the hypothesis of metal imbalances being the cause rather than a consequence of an ASD. Zinc supplementation reduced ASD symptoms in humans and other ASD animal models such as SH3 And Multiple Ankyrin Repeat Domains 3 (*Shank3)* and T-Box Brain Transcription Factor 1 (*Tbr1*) mice [159,160,161]. However, not all zinc-dependent activities will be similarly impacted by zinc deprivation. The degree of the zinc deficiency and the affinity of the proteins’ zinc-binding sites for zinc will determine the consequences of the deficiency or the presence of toxic metals competing with zinc. For example, only severe zinc deficiency or severe overload of toxic metals may impact high-affinity zinc-binding sites (e.g., zinc-finger domains) frequently present in transcription factors. This might explain why teratological effects are only seen in the case of severe zinc deficiency during pregnancy [162].

Zinc is essential for many vital processes that, if they are compromised, may give rise to the biological pathomechanism of ASDs. For example, free or weakly bound zinc ions regulate synaptic SHANK2 and SHANK3, NMDAR, or mitogen-activated protein kinase (MAPK). These proteins belong to the neurexin (NRXN)-neuroligin (NLGN)-SHANK and the phosphatidylinositol-3-kinase (PI3K)/Akt and mTOR signaling pathways, which have been linked to ASD [163]. Thus, both zinc status and genetic mutations affected the pathways that seem critical in the etiology of ASD. In turn, any environmental risk factors for ASD that impact zinc homeostasis, such as the presence of toxic metals, will also be linked to these key synaptic ASD-associated signaling pathways.

**Figure 2:**
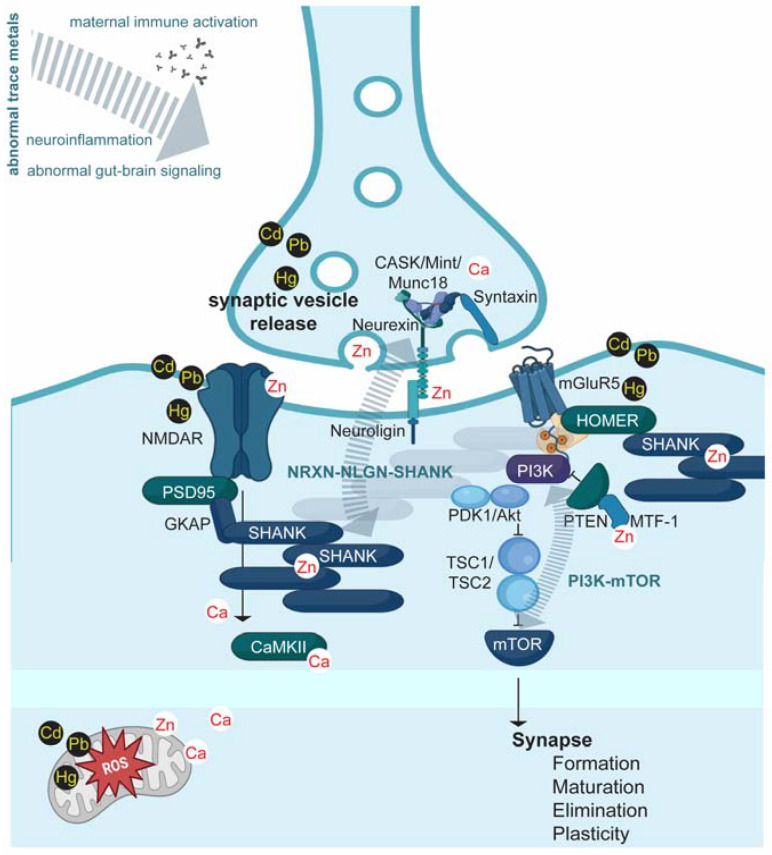
A model for the effects of metals and their contribution to ASD pathology. By considering metal profiles instead of isolated metals, the presence of heavy metals and deficiency in the essential metal zinc can be consolidated into one non-genetic risk factor for ASD. Together, these metal abnormalities will affect many biological processes in the body. However, direct and indirect effects (i.e., through altered gut-brain signaling, inflammatory cytokines, autoantibodies) on the NLGN-NRXN-SHANK and mTOR/PI3K pathway have been reported, which links this non-genetic risk factor to well-described pathomechanisms of genetic and other non-genetic factors, further consolidating multiple risk factors in one ASD-linked mechanism. Research has shown that both synaptic NLGN-NRXN-SHANK and mTOR/PI3K pathway activation is modified by zinc and calcium and affected through the interactions of toxic metals with NMDARs, mGluR5, and proteins mediating presynaptic vesicle release. The possible interaction of the metals with pathway proteins is indicated in the figure.

There is an emerging concept where parallel changes in other metal ions accompany changes in one metal. This connection between particular metals is also evident in the resulting cellular and behavioral pathologies. It is, therefore, plausible that, rather than a change in the one trace metal, a unique and possibly characteristic metal profile (metallome) is linked to behavioral traits associated with ASD (Figure 2). By considering metal profiles instead of isolated metals, the presence of heavy metals and zinc deficiency during pregnancy can be consolidated into one non-genetic risk factor for ASD. Thus, studies investigating metals in ASD should consider measuring specific toxic and essential metals. For example, the presence of a toxic metal may be linked to ASD pathology without the presence of measurable zinc deficiency by interfering with zinc signaling on the cellular level. In contrast, zinc deficiency could exacerbate the effects of relatively normal levels of toxic metals. Thus, there is a need for more metallomics studies in ASD research on the organism, tissue, cellular, and subcellular levels. 

In addition, studies in animals and concerning other diseases with complex etiologies bring hope that identifying particular metal-ASD crosstalk may be improved with the analytical strategies referred to as metallomics and ionomics supported by speciation analyses [164]. Typically, studies into metal determinations in human tissues or fluids concern the measurements of the total element concentration. However, clinical chemistry and toxicology will benefit from understanding the actions and effects of the specific species of elements. Chemical species have been defined as the “specific forms of an element defined as to electronic or oxidation state, isotopic composition, and/or complex or molecular structure.” In contrast, chemical speciation has been defined as the “distribution of an element among defined chemical species in a system” [165]. The assessment of the isotopic ratio may supply more information than the total metal contents. For instance, the ratio of ^207^Pb to ^208^Pb in bone samples may be useful for identifying the sources of lead exposure, whereas determining total Pb in bone samples characterizes body burden [166]. Findings from such speciation studies may support the implementation of public health prevention and control measures.

In turn, the oxidation state of an element plays a vital role in determining the element’s absorption, excretion, membrane transport, and, therefore, toxicity [167]. Speciation is considered a significant determinant of the bioavailability of an element. The type of inorganic metal complexes and organometallic species is linked to different solubility, hydrophobicity, and lipophilicity, thus determining which substances can pass through the blood-brain or blood-fetal barrier. It is well recognized that the brain and fetus are particularly prone to toxic organometallic species, including organic derivatives of Hg, Pb, and As [168,169]. Therefore, speciation analysis is a precious source of helpful information in studying various pathologies [170,171,172]. Unfortunately, very little is known about metal speciation in ASD, and the detailed description of the molecular mechanisms of single-species toxicity in ASD remains largely unknown. One reason for the limited number of such studies may be the very low concentrations of the various chemical species. Another reason is the stability problem of the metal-bioligand complexes present in clinical samples [173]. Studying the composition of elemental species in the cell and their associated proteins, as well as characterization of low molecular mass element species and elemental concentrations, for instance, in individual neurons, are among the challenges of modern biomedicine, which may provide novel insights into the role of toxic and essential elements in ASD.

Furthermore, limited tools are available to study the levels or activity of some metals in vivo. However, ^63^Zn-zinc citrate was developed as a positron emission tomography (PET) imaging probe of zinc transport and used in a human study [174], and probes for less abundant essential metals such as ^64^Cu, and ^52^Mn may provide new tools to perform “PET metallomics” in vivo in the future [175]. Besides, studies aiming at imaging metals are frequently conducted in Alzheimer’s research but rarely in individuals with ASD.

## 5. Conclusions

ASD is identified by the occurrence of two defining characteristics present in people with ASD, notably social deficits and repetitive and stereotypical behaviors, despite hundreds of different candidate genes and several non-genetic factors related to ASD. Given that neurological processes give rise to behaviors, it is likely that all potential causes disrupt these underlying processes and that they exhibit convergence not only on a behavioral level but also at the molecular level. As a result, the question of what neuronal mechanism underlies the symptoms of ASD and how so many hereditary and non-genetic factors might contribute to the same process arises. Intriguingly, metals affect the critical molecular aspects of ASD, including oxidative stress, inflammation, synapse development, synaptic communication, brain connectivity, and gut-brain signaling. As a result, one could speculate that a primary pathology of ASD may be a trace metal imbalance, characterized by either the presence of toxic metals and/or the overload or lack of essential metals, particularly a lack of zinc during brain development (pregnancy). In turn, genetic mutations and other non-genetic factors may recapitulate this trace metal imbalance fully or partially, leading to similar symptoms. If this model holds true in future studies, research on the etiology, prevention, and treatment needs to consider biometal physiology and pathology much more than it currently does. In particular, zinc supplementation during pregnancy may prevent toxic trace metal-associated ASD risks.

Furthermore, since metals exhibit varied effects on different molecular targets, there is a continuous need for research on metals with particular reference to the speciation forms and the concentration of metals, as well as routes and duration of exposure.

## Figures and Tables

**Table 1:** Overview of examples of epidemiological studies investigating the association of toxic metals with ASD. The toxic metals discussed in this review are highlighted. Systematic reviews and meta-analyses are highlighted.

Sample	Participants	Finding in ASD Participants	Reference
Hair	32 autistic children and 32 controls (4.1 ± 0.8 years)	**Pb↑**, Cu↑, **Hg↓**, Zn↓	[15]
Mercury intoxication-associated urinary porphyrins	28 individuals with ASD	**Hg** **↑**	[17]
Hair	18 children with ASD (3.5 ± 1.1 years)	**Hg↑** levels significantly correlate with CARS^1^ scores	[19]
Urine	129 individuals with ASD (14.1 ± 1.4 years) and 86 controls (14.7 ± 1.2 years)	**Cd↑,** Mn↑ significantly correlate with CARS ^1^ scores	[20]
Blood	48 children with ASD (5.5 ± 2.1 years)	19% had a Pb level of concern (greater than 0.1 μmol/L)	[21]
Hair	40 ASD boys and 40 controls (4.2 ± 2.2 years)	Sb↔, U↑, As↔, Be↔, **Hg↑**, **Cd↔, Pb↑,** Al↔	[22]
Hair	1967 children with ASD	Zn↓, Mg↓, Al↑, **Cd↑, Pb↑, Hg↑**, As↑	[23,24]
Blood, urine, hair	17 ASD children (11.5 ± 3.2 years) and 20 controls (10.4 ± 3.2 years)	**Hg↔, Pb↔,** Al↔, **Cd↔**	[25]
Teeth	15 ASD children (6.1 ± 2.2 years) and 11 controls (7 ± 1.7 years)	**Hg↑, Pb↔,** Zn↔	[26]
Hair, urine	25 ASD children (5.3 ± 1.9 years) and 25 controls (6.3 ± 2.3 years)	Hair: of 39 elements: As↑, **Cd↑**, Ba↑, Ce↓, **Hg↑, Pb↑,** Zn↓, Mg↓ Urine: of 39 elements: **Cd↓,** Al↑, Ba↑, Ce↓, **Hg↑, Pb↑,** Cu↑	[27]
Blood erythrocytes	83 ASD children (7.3 ± 3.7 years) and 89 controls (11.4 ± 2.2 years)	**Hg** **↑**	[28]
Mercury intoxication-associated urinary porphyrins	106 ASD children (mean 6.4 years) and 12 controls (mean 7.4 years)	**Hg** **↑**	[29]
Hair	65 ASD children (8.8 ± 0.5 years) and 80 controls (7.2 ± 0.7 years)	of 20 metals: **Hg↑, Pb↑,** As↑, Sb↑, **Cd↑**, Ca↓, Cu↓, Cr↓, Mn↓, Mg↓, Fe↓, Co↓, Se↑	[30]
Hair	27 ASD children (5.3 ± 1.5 years) and 27 controls (5.5 ± 1.4 years)	**Pb↑**, Al↑, Si↑, Mo↑, V↑, Cr↑, **Cd↑**, Co↑, Ni↑, B↑, Ba↑, Mg↑, Ca↓, Zn↓, Fe↑, Cu↓	[31]
Hair	30 ASD children (5.3 ± 1.6 years) and 30 controls (5.1 ± 1.5 years)	Ca↓, **Pb↑,** As↑	[32]
7	478 mother-child pairs	Gestational As↔, **Cd↑**, **Pb↑,** Mn↔, Hg↔	[33]
Teeth	80 ASD children and 113 controls (including twins)	Altered Zn-Cu cycles	[34]
Teeth	20 ASD discordant twin pairs, 12 ASD concordant twin pairs, 44 Non-ASD twin pairs	**Pb↑,** Mn↓, Zn↓, Mn and Pb significantly correlate with ADOS-2 and SRS-2 scores ^2^	[35]
Blood	2473 children	**Pb↑,** at 7–8 years significantly correlate with ASSQ and SRS ^3^	[36]
Hair	175 ASD children and 211 controls (including twins)	Zn, Li, Cu dysregulation	[37]
Hair, blood, urine, teeth	Meta-analysis of 48 studies with (ASD/control) samples: Sb (181/185) As (561/583) Cd (628/645) Pb (1508/1424) Mn (598/656) Hg (2246/2120) Ni (271/305)	As↔, **Cd↔**, Ni↔, **Pb↑** (hair, blood, erythrocyte), **Hg↑** (erythrocyte), Mn↔, Sb↑ (hair)	[38]
Hair, blood, urine	Meta-analysis of 18 studies for Al, 18 for Cd, and 23 for Hg with (ASD/control) samples: Al (1246/1199) Cd (1029/1030) Hg (1347/1348)	**Hg↑** (hair, blood, urine), Al↑ (hair, urine), **Cd↓** (hair, urine)	[39]

^1^ CARS: Childhood Autism Rating Scale. ^2^ ADOS-2: Autism Diagnostic Observation Schedule Second Edition, SRS-2: Social Responsiveness Scale Second Edition. ^3^ ASSQ: Autism Spectrum Screening Questionnaire, SRS: Social Responsiveness Scale.

## Data Availability

Not applicable.
